# Cognitive Neuroscience Approaches to Understanding Behavior Change in Alcohol Use Disorder Treatments

**DOI:** 10.35946/arcr.v37.1.03

**Published:** 2015

**Authors:** Nasir H. Naqvi, Jon Morgenstern

**Affiliations:** Nasir H. Naqvi, M.D., Ph.D., is assistant professor of clinical psychiatry in the Division on Substance Abuse, in the Department of Psychiatry at Columbia University, New York, New York.; Jon Morgenstern, Ph.D., is professor of psychiatry and director of addiction treatment in the Department of Psychiatry at North Shore-Long Island Jewish Health System, Long Island, New York.

**Keywords:** Alcohol use, abuse, and dependence, alcohol use disorder, neuroscience, cognitive neuroscience, brain, cognition, neural mechanisms, pathophysiology, behavior change, behavioral intervention, relapse, abstinence, treatment

## Abstract

Researchers have begun to apply cognitive neuroscience concepts and methods to study behavior change mechanisms in alcohol use disorder (AUD) treatments. This review begins with an examination of the current state of treatment mechanisms research using clinical and social psychological approaches. It then summarizes what is currently understood about the pathophysiology of addiction from a cognitive neuroscience perspective. Finally, it reviews recent efforts to use cognitive neuroscience approaches to understand the neural mechanisms of behavior change in AUD, including studies that use neural functioning to predict relapse and abstinence; studies examining neural mechanisms that operate in current evidence-based behavioral interventions for AUD; as well as research on novel behavioral interventions that are being derived from our emerging understanding of the neural and cognitive mechanisms of behavior change in AUD. The article highlights how the regulation of sub-cortical regions involved in alcohol incentive motivation by prefrontal cortical regions involved in cognitive control may be a core mechanism that plays a role in these varied forms of behavior change in AUD. We also lay out a multilevel framework for integrating cognitive neuroscience approaches with more traditional methods for examining AUD treatment mechanisms.

Understanding the mechanisms that underlie recovery from alcohol use disorder (AUD) is critical to advancing AUD treatment science (Huebner and Tonigan 2007; National Institute on Alcohol Abuse and Alcoholism [NIAAA] 2009). Scientific progress over the last three decades has led to the development of a number of effective behavioral and pharmacological AUD interventions (Dutra et al. 2008). However, even evidence-based treatments are only modestly effective. For example, reported rates of nonresponse to treatment interventions in major AUD treatment studies have ranged from 30 percent to 85 percent (Anton 2006; Johnson et al. 2007; McKay 2009; Project MATCH Research Group 1997). There is a general consensus that improving AUD behavioral intervention outcomes requires an understanding of the mechanisms that underlie behavior change in effective treatments (Magill and Longabaugh 2013; Morgenstern and McKay 2007). Thus, building a strong foundation for AUD treatment science includes answering the question of how, not just whether, a treatment is effective (Kazdin 2007).

To date, research on the mechanisms of effective AUD treatments that underlie behavior change have made limited progress, suggesting the need for major revisions in the theory and methods used for this work. Cognitive neuroscience may provide the tools for those revisions. Indeed, the pathophysiological processes that maintain AUD, such as craving, relapse, and withdrawal, are increasingly being understood in terms of the functioning of specific neural systems. As such, any psychosocial treatment for AUD that effectively changes behavior must interact at some level with these processes and, therefore, must influence these same neural systems. This article will review what cognitive neuroscience can tell us about the neural bases of AUD and the mechanisms by which psychosocial treatments may function to elicit behavior change in AUD patients.

## Psychosocial Treatment Mechanisms Research in AUD

There is a relatively large research literature on AUD behavioral treatment mechanisms (Huebner and Tonigan 2007; Longabaugh et al. 2013). This research largely represents an extension of assumptions and methods used to test treatment efficacy (Kazdin and Nock 2003; Morgenstern and McKay 2007; Wampold 2001). It has tested the treatment theories that guide evidence-based treatments using a set of mediation analysis procedures embedded within a clinical trials framework (Nock 2007). Stated succinctly, treatment theories postulate that the treatments work via some unique ingredient, often referred to as a specific effect—that is not present in other treatments (Morgenstern and McKay 2007). For example, theories postulate that motivational interviewing (MI) increases patients’ motivation to change their behavior (Miller and Rose 2009) and that neither a weak control condition like psychoeducation nor even a bona fide effective treatment like 12-step facilitation affects a patient’s motivation to change (Slaymaker and Sheehan 2013). Unfortunately, reviews of this literature generally conclude that there is limited support for most AUD treatment theories (Apodaca and Longabaugh 2009; Morgenstern and McKay 2007; Longabaugh et al. 2013). Indeed, most effective evidence-based AUD behavioral interventions yield equivalent outcomes even among subgroups where one would expect to find a difference. For example, MI typically has not proven superior to other AUD treatments among individuals with low motivation to change (Morgenstern and McKay 2007).

Even in instances where tests do not involve comparing treatments, it has often been difficult to establish seemingly straightforward links between treatment mediators and outcome. For example, Kelly and colleagues (2014) examined whether changes in peer networks mediated improved outcomes in 12-step treatment for young adults. Findings indicated that peer networks changed in the expected direction: posttreatment participants had fewer friends who used substances and more friends who abstained. Both greater affiliation with self-help organizations and changes in peer networks predicted improved outcome. However, contrary to prediction, the link between greater self-help affiliation and improved outcome was not mediated by changes in social networks. The authors concluded that more needs to be understood about how affiliation with self-help works to improve outcomes among youth with AUD.

It is important to note that some AUD treatment mediation studies have yielded important positive findings. For example, Moyers and colleagues (2009) found that improved outcomes in MI were mediated by increases in client motivational statements during treatment sessions. In addition, studies have consistently found that expected mediators such as motivation to change, self-efficacy, and social support for abstinence predict treatment outcome as well as improve during treatment, even though support for full mediation or specific effects generally has been absent. Overall, mediation analysis research has yielded less insight than expected about how AUD behavioral treatments work (Longabaugh et al. 2013). Given the relatively limited progress to date, it seems likely that major revisions in the theory and methods used to understand mechanisms of behavior change in AUD will be needed to advance this critical area of inquiry.

A major challenge to improving the informative value of AUD treatment mechanisms research is identifying the right measures to index the psychological processes that are hypothesized to mediate behavior change. Most of the conceptual frameworks and methods used to examine AUD treatment processes have not been revised to incorporate recent major conceptual and methodological advances for understanding the motivational, cognitive, affective, and, ultimately, neural processes that promote behavior change (Morgenstern et al. 2013). For example, constructs such as “motivation for change,” “peer networks,” or “coping skills” are very complex, and self-report measures designed to index them may encompass multiple psychological processes, some of which may relate to behavior change and others which may not. Furthermore, behavior change may depend upon psychological processes that are largely outside of conscious awareness and therefore not accessible by self-report measures. Moreover, such constructs may be difficult to relate to the underlying pathophysiology of addiction, which is understood increasingly in terms of highly specific affective, motivational, cognitive and neural processes. Cognitive neuroscience may hold the key to allowing researchers to use all of the processes to examine psychosocial treatment mechanisms.

## Why Use Cognitive Neuroscience Approaches?

There are several reasons why understanding psychosocial treatment mechanisms at the neural level will be critical for advancing AUD treatment. Any psychosocial treatments for AUD that are effective at changing behavior must interact at some level with the pathophysiological processes that maintain AUD, which themselves are being understood increasingly in terms of the functioning of specific neural systems. Indeed, identifying neural systems that play a role in behavior change in psychosocial treatments can help researchers hone current treatments and develop more effective ones. For example, it can facilitate more effective integration of behavioral treatments with medications, a goal that so far has proven elusive using purely clinical approaches (Combine Study Research Group 2006). In addition, measuring the functioning of brain systems involved in behavior change in a given treatment, especially when combined with genetic biomarkers, may be used to identify patients who are likely to respond to that treatment, another goal that has been elusive using purely clinical approaches (Project MATCH Research Group 1997). Other mental disorders that commonly co-occur with AUD, such as mood and anxiety disorders, also are now being understood in terms of the functioning of specific neural systems.

Among neuroscience approaches, cognitive neuroscience approaches have the most value for understanding psychosocial treatment mechanisms. Cognitive neuroscience approaches include a number of different methods aimed at understanding the relationship between relatively complex behaviors such as memory, attention, language, emotion and decisionmaking, and the structure and function of large-scale neural systems over relatively brief time periods (seconds). At a pragmatic level, cognitive neuroscience methods, such as structural and functional magnetic resonance imaging, allow for the noninvasive study of neural functioning in human subjects, which is critical in patient-oriented translational research. Also, compared with molecular or cellular approaches, the constructs addressed by cognitive neuroscience are nearer to the clinical phenomenology of AUD, as well as to the psychological constructs that have thus far been used to explain mechanisms of behavior change in AUD treatment.

Although cognitive neuroscience approaches may address certain clinically relevant questions that may improve the efficacy of psychosocial treatments, there is nothing inherently more valid or true about the neural level of understanding treatment mechanisms. A framework that integrates across multiple levels of analysis—social, interpersonal, behavioral, cognitive, and neural—will ultimately yield the most clinically useful understanding of behavior change. This would bring AUD research in line with the overall shift in mental health research to understand mental disorders and their treatments using a multilevel framework that includes neuroscience approaches (National Institute of Mental Health 2013).

## Neurocognitive Models of Addiction Pathophysiology

Arguably, more is known about the pathophysiology of AUD and other substance use disorders than of any other mental disorders. This is in large measure attributed to the development of highly valid animal models of drug and alcohol addiction that mimic the basic elements of human addiction, including drug self-administration, conditioned-place preference, and cued relapse. Researchers have coupled these animal models with invasive methods for measuring and manipulating neural function with a high degree of spatial and temporal localization in order to provide a detailed picture of the neural mechanisms that maintain addiction. The consensus that has emerged from this extensive body of work, reviewed at length elsewhere (Everitt and Robbins 2005; Koob and Le Moal 2001; Robinson and Berridge 2008), is that drugs and alcohol trigger dopamine-induced sensitization within incentive neural systems, in particular the ventral striatum, which normally motivate and guide the seeking of natural rewards but, after being sensitized, come to motivate and guide the seeking of drugs and alcohol.

In parallel with this animal literature, a large number of functional imaging studies in patients with substance use disorders have revealed neural systems whose activity is increased by exposure to drug and alcohol cues. Schacht and colleagues (2013) conducted a recent meta-analysis of functional magnetic resonance imaging (fMRI) studies in which AUD patients were exposed to alcohol-related cues. Their analysis showed that, consistent with animal models, alcohol cues reliably elicit neural activation in the ventral striatum. It also showed that alcohol cues elicit activation in cortical regions involved in decisionmaking, cognitive control, and emotional experience, such as the ventromedial prefrontal cortex, the anterior cingulate cortex, and the insula. Importantly, the analysis found that the ventral striatum was the region in which activity was most consistently related to behavioral and self-report measures of alcohol seeking, such as craving, and in which treatment most consistently reduced activity.

More recent work has examined the role of prefrontal cortical systems in various inhibitory, cognitive control, and decisionmaking functions that moderate or shape alcohol-seeking motivation in the service of long-term goals and the avoidance of negative consequences. A number of studies have shown that AUD is associated with structural and functional abnormalities in the prefrontal cortex (Goldstein et al. 2004; Volkow et al. 1994), along with neuropsychological impairments in a variety of executive functions mediated by the prefrontal cortex (Sullivan et al. 1993, 1997). Bechara and colleagues (2000), for example, have found a critical role for the ventromedial prefrontal cortex in the successful performance of behavioral tasks that require the forgoing of short-term, but certain, rewards to avoid long-term, but uncertain, negative consequences. Subsequently, they demonstrated that AUD patients show impairments on these same behavioral tasks, similar to impairments seen in patients with ventromedial prefrontal cortex damage (Bechara and Damasio 2002; Bechara et al. 2002). The decisions in these tasks resemble an AUD patient’s decision to abstain or relapse, which is a decision to obtain a short-term reward (alcohol) without regard to a variety of uncertain, long-term negative consequences. Additionally, fMRI studies have linked dysfunction in the dorsolateral prefrontal cortex to impaired inhibitory control in AUD (Li et al. 2009). One study (Field et al. 2007) has linked AUD with impairments in delayed discounting and executive attention functions, both of which depend upon prefrontal cortical regions. A more recent study (Naqvi et al. 2015) finds that, compared with social drinkers, AUD patients are less able to reduce cue-induced craving by thinking about long-term negative consequences of alcohol use. This ability is a cognitive regulation function that fMRI studies in cigarette smokers show depends upon functional interaction between the dorsolateral prefrontal cortex and the ventral striatum (Kober et al. 2010).

Together, this work suggests that AUD is maintained by the interaction of two neural adaptations that arise as a result of chronic alcohol use:

- The dopamine-induced sensitization of the ventral striatum to alcohol and alcohol-related cues, leading to enhanced emotional and behavioral reactivity to these stimuli; and
- Impairments in prefrontal cognitive control functions, leading to an inability to regulate emotional and behavioral hyperreactivity to alcohol and alcohol-related cues that are driven by a sensitized ventral striatum.

These neural adaptations make it difficult for AUD patients to control alcohol use in the face of negative consequences, a hallmark of AUD. If this model is correct, then effective treatments for AUD should either directly downmodulate the ventral striatum reactivity to alcohol and alcohol-related cues, or they should enhance the pre-frontal cortex’s ability to regulate ventral striatal reactivity to alcohol and alcohol-related cues according to long-term goals and consequences.

## Neurocognitive Predictors of Relapse

If AUD patients remain abstinent after they stop drinking, it suggests that the behavior change mechanisms of their treatment worked. Conversely, if they relapse after a period of abstinence, it suggests that the same behavior change mechanisms failed. Thus, it may be possible to infer mechanisms of behavior change by identifying neural measures that predict relapse and abstinence. One of the first studies to do this, by Wrase and colleagues (2008), measured regional brain volumes in several reward-related brain regions in detoxified AUD patients. They found that the volume of the amygdala was lower in patients who relapsed to heavy drinking by 6 months, compared with those who abstained. Subsequently, Cardenas and colleagues (2011; Durazzo et al. 2011) showed that, compared with patients who abstained, patients who relapsed by 8 months posttreatment had relatively smaller total volume in the orbitofrontal cortex. Similarly, Rando and colleagues (2011) showed that patients with a smaller volume of gray matter in medial prefrontal regions, including the anterior cingulate cortex, relapsed more quickly and were more likely to drink heavily during relapse than patients with larger gray-matter volumes. What is not clear from these studies is whether a reduction in volume represents a loss of function, which would tend to increase relapse risk in the case of prefrontal cognitive control systems that regulate alcohol seeking, or whether the reductions represent a gain of function, which would tend to increase relapse risk in the case of incentive motivational systems that promote alcohol seeking.

These limitations may be addressed by functional imaging studies that examine how neural activity measured under various conditions predicts relapse. Several of these studies have been completed to date:

- Seo and colleagues (2013) measured neural activity during alcohol cue exposure, stressful imagery, and neutral imagery. They found that activity in the ventromedial prefrontal cortex and anterior cingulate cortex during neutral imagery predicted relapse within 3 months.
- In a small study, Braus and colleagues (2001) showed that alcohol cue–elicited activity in the ventral putamen predicted relapse within 3 months.
- Grusser and colleagues (2004) showed that alcohol cue–elicited activity in the putamen, anterior cingulate, and adjacent medial prefrontal cortex predicted relapse at 3 months.
- Heinz and colleagues (2007) failed to show a correlation between alcohol cue–elicited neural activity and relapse within 6 months but did show that neural activity elicited by positive emotional pictures within the thalamus and ventral striatum predicted abstinence.
- Camchong and colleagues (2013) showed that lower resting-state connectivity between “reward” and “executive control” regions during early abstinence predicted relapse within 6 months. They also found that resting-state connectivity between these systems was negatively correlated with poor inhibitory control in an affective go/no-go task.

Many of these functional imaging studies did not address patients’ engagement in informal treatments such as 12-step groups during the follow-up period. This limitation makes it unclear whether neural activity was predictive of “intrinsic” abstinence capabilities, or of the capacity to respond to these informal treatments. That said, together, these structural and functional imaging studies point toward neural systems that promote abstinence that already has been initiated. As such, they may not be generalizable to understanding the neural mechanisms by which actively drinking AUD patients reduce their alcohol use. This may bear upon the distinction between treatments intended to prevent relapse and treatments intended to initiate abstinence or to moderate alcohol use. Moreover, it is not clear whether results of studies examining predictors of abstinence and relapse in nontreatment samples can even be generalized to understand behavior change that results from effective treatments. This will require studies that examine neural functioning in treatment-seeking AUD patients both prior to and after completing treatment.

## Neurocognitive Mechanisms of Existing, Evidence-Based AUD Treatments

A small number of studies have attempted to examine the specific neurocognitive mechanisms by which existing effective behavioral interventions change behavior, a concern that is central to mechanisms of behavior change initiation (MOBC) research (NIAAA 2009). In one study, Vollstät-Klein and colleagues (2011) used fMRI to examine changes in neural activity elicited by alcohol-related cues both before and after participants received nine sessions of cue-exposure treatment (CET), which was added to supportive outpatient treatment. The researchers compared these patients with patients who received supportive outpatient treatment alone. They found that patients receiving CET showed a greater reduction in cue-elicited activity in the ventral and dorsal striatum, the anterior cingulate cortex, the precentral gyrus, the insula, and several prefrontal regions. This finding is consistent with a reduction in the rewarding interoceptive effects of alcohol as a result of CET.

DeVito and colleagues (2012) used fMRI to examine changes in neural activity related to the Stroop color–word interference task, which engages cognitive control and executive attention functions, in patients with substance use disorders that included AUD. Patients performed the Stroop task during fMRI both before and after receiving treatment. Half of the patients received treatment as usual from an outpatient drug treatment program along with 8 weeks of biweekly computerized cognitive behavioral therapy (CBT). The other half only received treatment as usual. Study authors found that patients receiving CBT improved their performance on the Stroop task and had decreased task-related activity in the anterior cingulate cortex (ACC), inferior frontal gyrus, and the midbrain. This is consistent with the theory that CBT improves general cognitive control functions. The study did not examine whether CBT changed neural activity related to alcohol-specific cognitive control functions, such as performance on an alcohol-specific Stroop task or cognitive regulation of alcohol craving, which would speak more specifically to the mechanisms of changing alcohol use behavior, as opposed to general self-regulatory mechanisms. Furthermore, this study did not examine AUD specifically but rather grouped patients with AUD with patients with other substance use disorders.

In another fMRI study, Feldstein Ewing and colleagues (2011) compared neural responses with alcohol cues during exposure to “change talk” and “counterchange talk,” which are linguistic/semantic constructs hypothesized to mediate behavior change in MI. Study participants were AUD patients seeking treatment. The study found that exposing patients to alcohol-related cues while they listened to counterchange talk elicited activity in the ventral striatum, orbitofrontal cortex, and insula, whereas none of these areas showed any activity during change talk. These regions all play a role in representing the incentive value of rewards. This suggests that change talk may downmodulate the neural representations of the incentive value of alcohol-related cues. The study did not examine how these responses changed over the course of MI treatment, which would be necessary to infer whether this mechanism actually plays a role in this particular treatment.

These studies are important first steps; however, they possess a number of limitations. For example, none of them reported drinking outcomes after the interventions, which limits the ability to infer whether changes in neural functioning due to the interventions drive behavior change. Also, the control interventions were not themselves effective treatments that were missing only the hypothesized behavior change mechanism. This is important because existing evidence-based AUD treatments are complex, with multiple psychological components, many of which potentially affect behavior. This makes it necessary to examine neural mechanisms of behavior in existing treatments in a “top-down” fashion by decomposing complex intervention-specific constructs, such as change talk and coping skills into specific neurocognitive functions, such as reversal learning, cognitive control, emotion regulation, and response inhibition, both as they relate to alcohol and as they relate to general reward functions.

## Novel AUD Treatments Derived From Neurocognitive Mechanisms

An alternative approach to understanding behavior change in AUD involves constructing novel interventions based upon our current understanding of the neurocognitive mechanisms of AUD pathophysiology and behavior change. As discussed above, AUD is associated with impairments in a number of executive functions that require regulation of subcortical reward-related and automatic processes by prefrontal regions, including working memory, inhibitory control, reward learning, and craving regulation. Thus, interventions targeted at remediating these impairments should lead to reductions in alcohol use behavior. This provides both a new set of effective treatments and also indirectly tests hypotheses about the role of cognitive functions that are being remediated and, by extension, their neural substrates, in behavior change.

In a study by Houben and colleagues (2011*b*), non–treatment-seeking heavy drinkers completed 25 daily sessions of general working-memory training, including tasks designed to improve digit span, letter span, and visual-spatial working memory, all with progressively increasing difficulty. A heavy-drinking control group performed similar tasks that did not increase in difficulty.

Participants in the active intervention group had improved working-memory function and, more importantly, significantly reduced the number of drinks they drank per week, compared with participants in the control group. This effect persisted for more than a month. The researchers also collected data on participant performance on an implicit alcohol association test, which measures the automaticity of processing alcohol-related information. They found that changes in working-memory capacity mediated the effects of working-memory training on reduction in alcohol use and that baseline performance on the implicit association test moderated this relationship. These findings provide circumstantial evidence that working-memory training reduced drinking by increasing control over automatic alcohol-related processing.

In another study, Houben and colleagues (2011*a*) examined the effect of a different cognitive task on non–treatment-seeking heavy drinkers. In a single session, one group of participants learned to provide “go” responses to non–alcohol-related cues and “no-go” responses to alcohol-related cues. Another heavy drinking group completed a version of the task requiring “go” responses to alcohol cues and “no-go” responses to nonalcohol cues. The researchers found that subjects in the no-go alcohol group significantly reduced their drinking in the week after the task, whereas subjects in the go alcohol group increased their drinking. Performance on this kind of go/no-go paradigm depends upon inhibitory control as well as reward-learning functions, suggesting that such functions may play a role in behavior change in AUD. However, this study did not provide a direct test of this model.

Both of these studies were relatively small and were undertaken in non–treatment-seeking heavy drinkers, as opposed to treatment-seeking patients diagnosed with AUD. Therefore, it is not known if these interventions would have similar effects in more severe, treatment-seeking AUD populations, who generally have more severe drinking problems and are likely to have a higher level of dysfunction in the neurocognitive functions being addressed by these interventions. It also is possible that the effects of these interventions were small, compared with potential effects of entering into a formal treatment with a high level of motivation for change, as is the case with many treatment seekers.

A larger study by Wiers and colleagues (2011) addressed these limitations. The study examined the effect of cognitive-bias modification (CBM) given to AUD patients prior to entering inpatient rehabilitation. CBM involved training patients to push a joystick away (an avoidance movement) whenever they saw an alcohol cue. This intervention is similar to the go/no-go task in that it involves repeatedly assigning a negative value (in this case a movement with intrinsic negative valence) to alcohol. Participants in the control groups received either no training or a training condition in which they had to make equal numbers of avoidance movements to alcohol cues and nonalcohol cues. The researchers followed patients for a year after they completed inpatient rehabilitation. The results showed that patients who received CBM prior to entering inpatient rehabilitation were somewhat less likely to relapse. And although the effect was just below the threshold for statistical significance, it provides circumstantial evidence that such implicit forms of reappraisal of alcohol’s value may affect behavior change.

## Summary and Limitations of Cognitive Neuroscience Approaches

**Figure 1:**
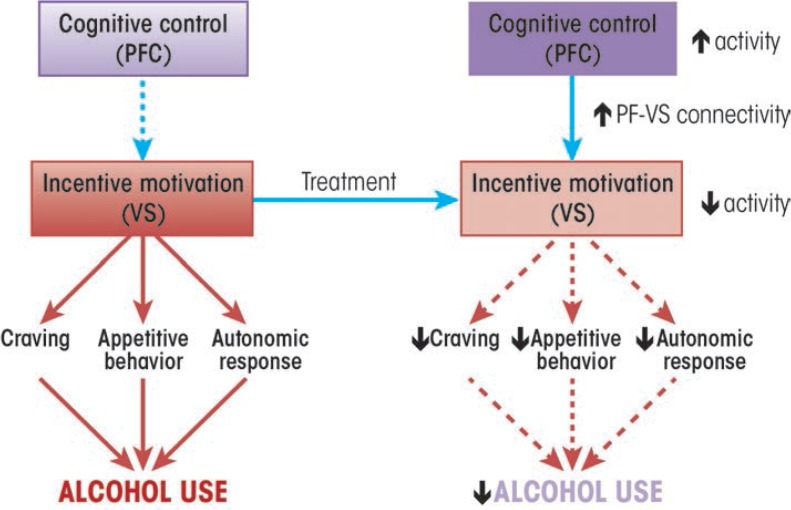
A potential common mechanism for alcohol use disorder (AUD) treatments. A number of studies suggest that AUD treatments elicit behavior change by increasing the regulation of brain regions that mediate incentive motivation, such as the ventral striatum, by prefrontal cortical regions that mediate cognitive control. Arrows denote expected changes in specific neural, behavioral, psychophysiological and clinical outcome measures, given this hypothesized treatment mechanism. PFC = prefrontal cortex. VS = ventral striatum.

A theme that emerges from the disparate lines of research reviewed here is that effective treatments for AUD serve to increase prefrontal cortex function and downmodulate the function of reward systems, especially the ventral striatum. Given the role of functional interactions between the prefrontal cortex and the ventral striatum in a variety of self-regulation processes (Ochsner et al. 2012), it is likely that increased functional interaction between these regions may serve as a critical behavior change mechanism that is shared by a number of different effective psychosocial treatments. In other words, findings from cognitive neuroscience predict that effective treatments increase prefrontal cortical function, decrease ventral striatal function, and increase functional connectivity between these two regions, especially during the processing of alcohol-related information (figure 1). Although a number of the studies cited here provide circumstantial evidence for this mechanism, no studies have tested it directly.

Another important theme that emerges from this literature is whether behavior change mechanisms related to AUD are specific to alcohol use or more general cognitive changes. AUD is associated with deficits in a number of general cognitive functions, especially executive and cognitive control functions, as well as specific “gains of function,” with respect to the incentive and rewarding effects of alcohol and related cues. Thus, it is important to understand whether a given intervention changes alcohol use behavior because it influences general cognitive functions or because it influences functions that are specific to the processing of alcohol-related information. For example, it is possible that interventions aimed at reducing the incentive salience of alcohol cues, such as cue-exposure therapy, and interventions aimed at increasing the ability to specifically regulate this incentive salience, such as cognitive bias modification and cognitive regulation of craving, are mediated by the specific mechanism of prefrontal executive/cognitive control regions modulating the processing of alcohol’s incentive value by subcortical reward-related regions. Concurrently, interventions aimed more generally at improving prefrontal cortex functions, such as working-memory training, may facilitate the more specific interventions because these general functions play a part in alcohol-specific regulation functions.

Although cognitive neuroscience approaches provide a window into AUD treatment mechanisms that aligns with our current understanding of AUD pathophysiology, there are limitations to cognitive neuroscience approaches that affect the ability to infer AUD treatment mechanisms. A major limitation of all functional imaging studies is that they are essentially correlational. Merely showing that a given psychological process is associated with increased activity within a specific neural system does not by itself prove that this neural system is critically necessary for the psychological process. By extension, merely showing that neural activity within a brain system changes as a result of a treatment does not demonstrate that this treatment must affect this brain system to elicit behavior change. When examining disease pathophysiology, it is difficult to know whether differences between patients and healthy controls in brain structure and function play a causal role in disease pathology, whether they are merely parallel phenomena, or whether they pre-exist disease development. This issue may be addressed in prospective studies in at-risk individuals (see Ersche et al. 2012 for an example of this approach applied to structural brain abnormalities in addiction). Such limitations are not specific to AUD treatment research; they are inherent in all translational neuroimaging studies that aim to examine pathophysiology and treatment mechanisms.

## Future Directions

Using cognitive neuroscience approaches to study behavior change in psychosocial treatments for AUD is a young field. Future studies can address some of the current weaknesses of this field by integrating cognitive neuroscience approaches with the conceptual and methodological approaches that already have proven useful for examining AUD treatment mechanisms. The first step in such an approach is to identify specific cognitive, affective, and behavioral processes that are hypothesized to mediate behavior change in a given treatment. The next step is to operationalize these processes using relatively simple paradigms that can be implemented in functional imaging experiments. This also should include appropriate control tasks that are ideally the same as the experimental tasks, minus the psychological processes under study. There should be preliminary data showing which neural parameters (i.e., activity measures in specific brain systems, along with measures of connectivity between brain systems) are changed by this task, compared with the control task, and how this relates to behavioral measures acquired during the functional imaging experiments. There should also be a clear set of a priori hypotheses about which of these neural parameters relate to behavior change in the treatment and which do not. The clinical population should be well characterized using self-report measures of AUD severity and or psychological processes that have already been studied as mediators of behavior change in the treatment under study. Patients should be randomly assigned to receive the active treatment or an equally effective control treatment that is hypothesized to not depend upon the processes under study. Functional imaging data, along with self-report measures, should be acquired both prior to and then immediately following the treatments. Appropriate clinical outcome measures should be specified.

**Figure 2:**
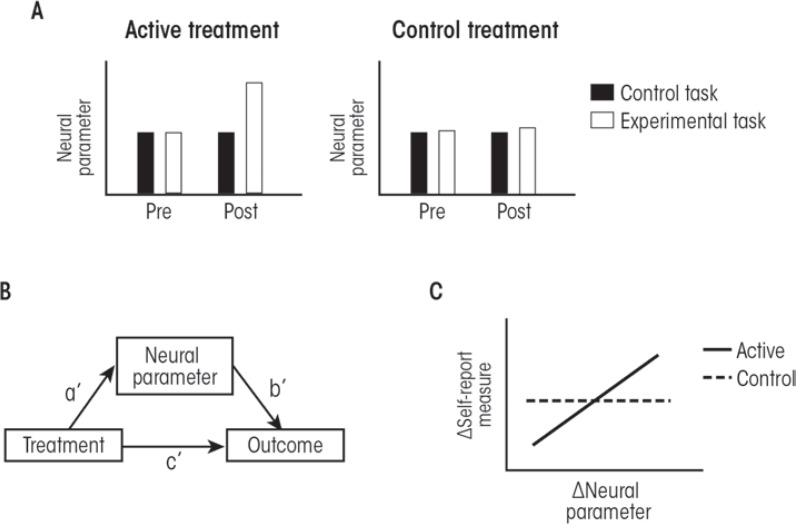
Predicted results from experiments directed at addressing the role of neural systems in alcohol use disorder (AUD) treatment mechanisms. **(A)** An active treatment should increase the neural parameters that index the functioning of these systems as it relates to a specific psychological process of interest (the experimental task). There should be no effect of the control treatment on these neural parameters. **(B)** The effects of a treatment on the neural parameter should mediate the effects of the treatment on clinical outcome. **(C)** Changes (Δ) in the neural parameters from pre- to posttreatment should correlate with corresponding changes in self-report measures that index psychological processes already known to drive behavior change.

What kind of results would be necessary to support the role for a specific neural system in the mechanism of a treatment? First, it would be necessary to show that the active treatment, but not the control treatment, changed the functioning of this neural system as it relates to the specific psychological process under study. Second, it would be necessary to show that the relationship between the treatment and the clinical outcome was statistically mediated by the effect of treatment on the functioning of this neural system. Third, it would be useful to relate changes in neural function from pre- to posttreatment to changes in self-report measures indexing psychological processes already known to mediate behavior change in the treatment. This would help to clarify whether the neural system plays a role in psychological processes already known to be involved in behavior change, or whether neural systems impact some other, as yet unknown, psychological processes that drive behavior change. This approach is illustrated in figure 2.

Once a neural system is identified as playing a role in behavior change in a specific treatment, additional studies can use “interventional” approaches, such as transcranial magnetic stimulation, to examine how noninvasively disrupting or enhancing the functioning of this neural system impedes or augments behavior change during the treatment. Additionally, researchers can add medications that are known to target this neural system to the treatment, and observe the effect on behavior change. Researchers also can seek out AUD patients who acquire brain damage in the neural system—for example from a stroke—and examine whether the brain damage reduces the efficacy of the treatment as a result of impairments in the psychological processes mediated by the damaged neural system. These approaches would provide direct tests of the role of the neural system and the psychological processes it mediates in behavior change, as opposed to the correlational evidence provided by functional neuroimaging.

Although such an approach attempts to relate changes in neural parameters acquired in functional imaging experiments to changes in behavior, it is important to note that the neural parameters by themselves do not constitute a mechanism. Rather, they are measurements of the functioning of specific neural systems that are involved in psychological processes that drive behavior change. In this way, the approach must integrate across multiple levels of analysis. Such an integrative approach does not place a higher value on neural measures compared with psychological or clinical measures. Instead, the approach depends on several levels of analysis in order to arrive at a coherent, clinically useful understanding of how currently effective treatments change behavior, one that can ultimately facilitate the development of novel, more effective treatments.
